# Tissue steroid levels in response to reduced testicular estrogen synthesis in the male pig, *Sus scrofa*

**DOI:** 10.1371/journal.pone.0215390

**Published:** 2019-04-15

**Authors:** Heidi Kucera, Birgit Puschner, Alan Conley, Trish Berger

**Affiliations:** 1 Department of Molecular Biosciences, School of Veterinary Medicine, University of California, Davis, CA, United States of America; 2 Department of Population Health and Reproduction, School of Veterinary Medicine, University of California, Davis, CA, United States of America; 3 Department of Animal Science, College of Agricultural and Environmental Sciences, University of California, Davis, CA, United States of America; Universite de Rouen, FRANCE

## Abstract

Production of steroid hormones is complex and dependent upon steroidogenic enzymes, cofactors, receptors, and transporters expressed within a tissue. Collectively, these factors create an environment for tissue-specific steroid hormone profiles and potentially tissue-specific responses to drug administration. Our objective was to assess steroid production, including sulfated steroid metabolites in the boar testis, prostate, and liver following inhibition of aromatase, the enzyme that converts androgen precursors to estrogens. Boars were treated with the aromatase inhibitor, letrozole from 11 to 16 weeks of age and littermate boars received the canola oil vehicle. Steroid profiles were evaluated in testes, prostate, and livers of 16, 20, and 40 week old boars using liquid chromatography/mass spectrometry. Testis, prostate, and liver had unique steroid profiles in vehicle-treated animals. Only C18 steroid hormones were altered by treatment with the aromatase inhibitor, letrozole; no significant differences were detected in any of the C19 or C21 steroids evaluated. Testis was the only tissue with significantly decreased free estrogens following treatment with the aromatase inhibitor; estrone and estradiol concentrations were lower (p < 0.05) in testes from 16, 20, and 40 week letrozole-treated boars. However, concentrations of the sulfated conjugates, estrone-sulfate and estradiol-sulfate, were significantly decreased (p<0.05) in 16 and 20 week boar testes, prostates, and livers from letrozole-treated boars. Hence, the distribution of estrogens between the free and conjugated forms was altered in a tissue-specific manner following inhibition of aromatase. The results suggest sulfated testicular estrogens are important estrogen precursors for the prostate, potentially enabling peripheral target tissues to synthesize free estrogens in the male pig.

## Introduction

To fully understand steroid hormone synthesis, regulation, and the mechanisms involved is challenging. Interplay and regulation of enzymes, cofactors, receptors, steroid hormones, and protein hormones within a single tissue are complex [1–10]. Recently, the role of steroid sulfates and the expression of sulfotransferase and sulfatase have been recognized as critical players in the regulation of steroid hormone production in a tissue [7, 11]. Quantitatively, the most important steroid hormone in the human circulation is dehydroepiandrostenedione-sulfate (DHEA-sulfate) with concentrations 100–10,000 times higher than circulating androgens and estrogens [12]. The classical theory was that sulfate conjugation was strictly for enhanced clearance of steroid hormones due to increased water solubility and inability to bind to steroid receptors [13]. More recently, increased biological half-life of sulfo-conjugates and desulfation as a path to free steroids are recognized as potential alternative roles [1, 2, 7, 14].

The boar is a distinctive model amongst male mammals because the testes produce high levels of circulating estrogens [15, 16]. The boar also possesses reproductive attributes such as large accessory sex glands and testes, large ejaculate volume, highly developed interstitial tissues in the testis, and the extensive steroid producing capabilities of the Leydig cells [15, 17]. To better understand the regulation and synthesis of steroid hormones, specifically estrogens, in the boar, we utilized an aromatase inhibitor, letrozole. Aromatase catalyzes the rate-limiting step in estrogen biosynthesis, the conversion of C19 androgens to C18 estrogens [18, 19]. Previously, our laboratory developed a letrozole-treated boar model to evaluate the influences of endogenous estrogens on the reproductive development of boars [20–25]. Although no effect of letrozole treatment on androgens had been detected in the prior work, effects of this inhibition of aromatase on less abundant androgens and other steroids in the testis and in other tissues remained unanswered. The questions of why the boar produces or requires the large amount of endogenous estrogens synthesized [26], and which mechanisms regulate this synthesis [27, 28] are not understood.

The potential roles of steroid sulfates, specifically DHEA-sulfate and estrogen sulfates, in steroid hormone production and distributions are intriguing, given the substantial amounts of sulfated steroid hormones in the boar [29, 30]. This study focuses on the interplay between free and conjugated steroid hormones in a tissue. Raeside et al. called for a more complete profile of steroid hormones in the boar testis, specifically estrogens [15] and the critical need to better understand local metabolism of steroid hormones within a tissue [31]. To address this critical gap, over 200 different C21, C19 and C18 steroids were assessed in testis, prostate, and liver at different developmental stages in boars treated with letrozole during the late juvenile interval. The multi-tissue approach in combination with a broad investigation of steroids and aromatase inhibition, revealed the complexity and unique role each organ has in steroid hormone synthesis. These data allow consideration of the roles of the testis, prostate, and liver in endogenous estrogen regulation and steroid hormone production.

## Materials and methods

### Animal treatment, tissue collection, and experimental design

Animals were treated humanely in strict accordance with protocols approved by the University of California, Davis Institutional Animal Care and Use Committee (#13398 and #16308). Avoidance of stress was a goal in administration of treatments and all efforts were made to eliminate suffering. Twenty-four boars were offspring from animals donated by PIC USA (Franklin, KY, USA) and semen donated by Genus plc (Hendersonville, TN, USA). Animal procedures have been detailed previously [22, 32]. Letrozole was a gift from Ciba-Geigy (Basel, Switzerland). Briefly, littermates (total of 24 boars) from five litters were randomly assigned to either vehicle treatment (n = 12) or letrozole treatment (n = 12). Between 11 to 16 weeks of age (prepubertal treatment), each boar received weekly oral administration of letrozole (0.1mg/kg body weight) or canola oil (vehicle treatment) mixed in 50 grams of feed. To collect tissue specimens, pigs were euthanized by electroshock and subsequent exsanguination at 16, 20, and 40 weeks of age. The 16 week age was selected since that is the time when sperm first appear in the epididymis, 20 weeks was selected since that age was associated with peak testicular testosterone [33] in this genetic background, and 40 weeks was considered a mature age to determine if prepubertal letrozole treatment reprogrammed testicular steroid concentrations. At each age, one boar treated with letrozole from four different litters and the corresponding littermate treated with vehicle provided tissue samples. Testis, prostate, and liver tissue samples were flash-frozen on dry ice and stored at -80°C until analysis. Only three liver samples were available to evaluate the 16 week letrozole-treated boars (n = 3).

### Quantification of steroids

Samples were extracted using a previously described modification [34] of a published method [35]. Briefly, ~200 mg of tissue were homogenized and extracted twice with cold methanol 1:2, weight: volume. After centrifugation, the methanol supernatants were removed and stored in -20°C. The remaining pellet was extracted twice with 1ml chloroform, centrifuged, and the supernatant collected. Both the methanol and chloroform extracts were combined and dried with a centrifugal concentrator. The residue was reconstituted in 125 μl of methanol, filtered through a 0.2 μm ultracentrifuge filter (Millipore Inc., St. Louis, MO, USA), and 7 μl injected into the UPLC/MS-MS.

The ultra-performance liquid chromatography–electrospray mass spectrometry (UPLC-ESI/MS/MS) system consisted of a Waters Acquity UPLC system (Waters Inc., Milford, MA, USA) coupled to a Waters Xevo-TQ mass spectrometer (Waters Inc., Milford, MA, USA). A detailed descriptions of the method used to quantify steroids has been described previously [34, 36]. Analytical data were processed by TargetLynx 4.1 software (Waters Inc., Milford, MA, USA).

### Tissue enzymes

Microsomes were prepared from tissue (from 16 week old animals) homogenized in 0.1M potassium phosphate buffer (pH 7.4) containing 20% glycerol as previously described [37]. Aromatase activity was determined using the tritiated water assay [38]. Steroid sulfatase (*STS*) gene expression was evaluated in tissues from 3 animals by qPCR using TCTGTCACGGGCATTCCATC as the forward primer and CGGAGTTAACGGGTCTGTCT as the reverse primer. Product size (89) of the PCR product was validated on an agarose gel. Efficiency was calculated at 98.6%, R^2^ for linearity was 0.99 and melt curves indicated a single product; these observations were validation of the qPCR. The reference gene was *RARS* [39].

### Statistical analysis

Statistical analysis and principal component analysis of steroid concentrations were performed using Origin software (OriginLab, Northampton, MA, USA). Tissue steroid concentrations were analyzed using a fixed model and one-way analysis of variance (ANOVA). Differences between means from vehicle and letrozole treatments at the same age were evaluated with Tukey’s honestly significant difference. Effects were considered statistically significant at P < 0.05. Data were also transformed to improve normality (log, square root, log(square root) or reciprocal transformations) and ANOVA rerun. The absence of significant treatment effects for the C19 and C21 steroids was the same for transformed and nontransformed data. Detected treatment effects on the C18 steroids were similar for the transformed and nontransformed data although P values did vary slightly. Results are presented as means (of the initial data) ± standard deviation, as a percentage of summed total steroids and as a percentage of each class (C21, C19 and C18). Principal component analysis was used to visualize differences among tissue type and treatment. Logged transformed data were imported into Origin software with values below the limit of quantitation input as one half of the limit of detection. Aromatase activity and gene expression were analyzed using lmer (mixed model with litter as a random factor) and aov (fixed model) functions in R statistical programs [40].

## Results

### Letrozole effects on C18, C19, and C21 steroids

**Fig 1:**
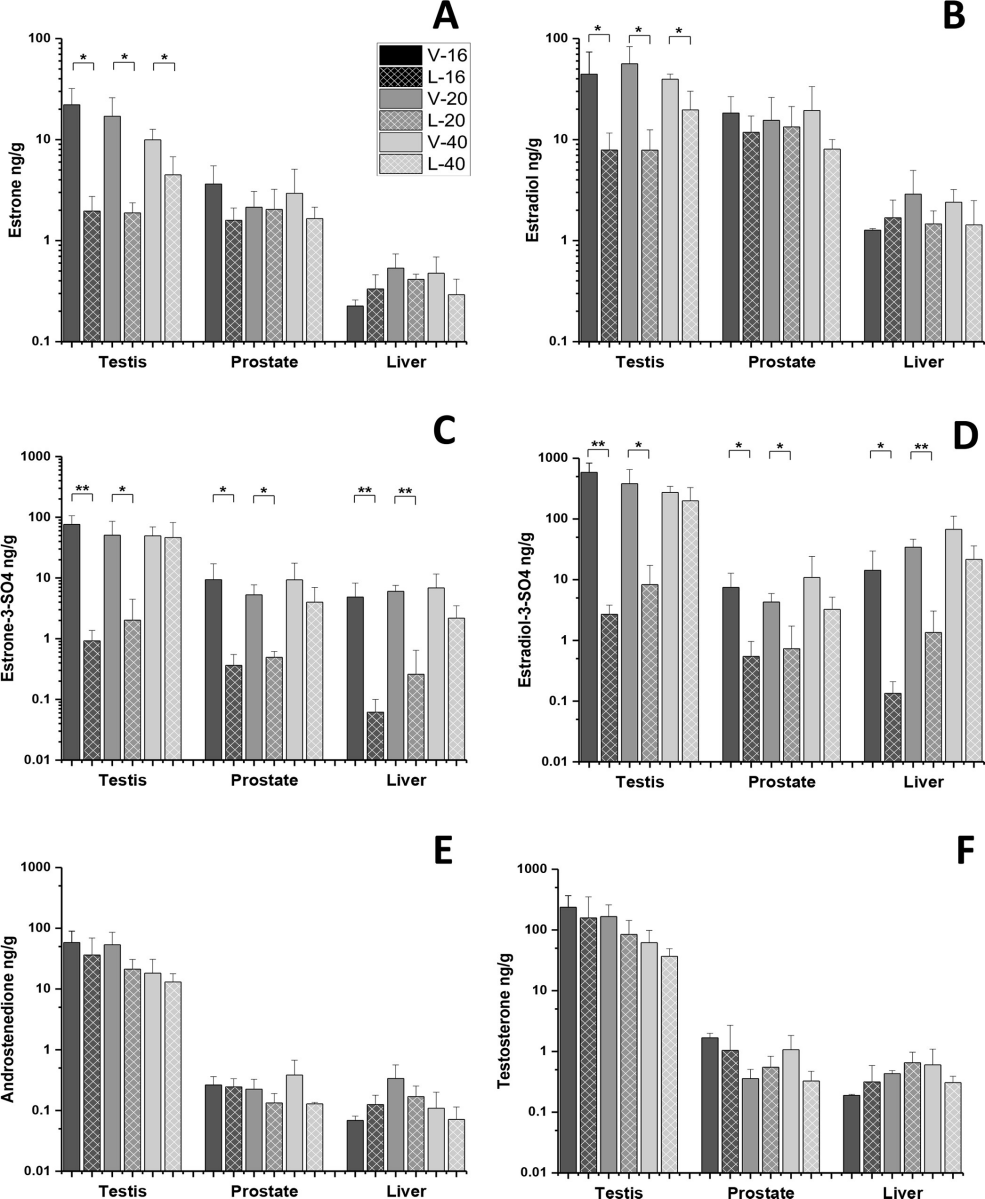
Letrozole induced changes in tissue concentrations (pg/g) of estrogens and androgens in the testis, prostate, and liver. Estrone (**A**) and estradiol (**B**) concentrations were decreased in the testis of 16, 20, and 40 week boars compared with vehicle-treated littermates. Estrone (**A**) and estradiol (**B**) concentrations in the prostate and liver were not affected by letrozole treatment. Estrone sulfate (**C**) and estradiol sulfate (**D**) concentrations were decreased compared with vehicle-treated littermates at week 16 and 20 in all tissues. The aromatase substrates, androstenedione (**E**) and testosterone (**F**), were not affected in any tissue by letrozole administration. * P < 0.05, ** P < 0.01.

Letrozole administration resulted in statistically significant decreases in estradiol and estrone concentrations in testes at all ages, while no concentration changes in free estrogens were observed in prostate or liver (Fig 1A and 1B). Sulfated conjugates of estradiol and estrone were significantly decreased in the testis, prostate, and liver from the 16 and 20 week letrozole-treated boars but not in the 40 week letrozole-treated boars (Fig 1C and `D). Interestingly, estradiol sulfate and estrone sulfate concentrations were similar or lower than their free forms in the testis, prostate, and liver tissue extracts from 16 and 20 week letrozole-treated boars but not in their vehicle-treated littermates (Table 1). Hence, letrozole treatment alters testicular distribution of C18 steroid hormones in 16 and 20 week boars, but free C18 steroid distributions are less affected in the prostate and liver (Table 1). Letrozole treatment did not alter any androgen in any of the three tissues at any time point (Fig 1E and 1F, Table 1) compared with vehicle-treated littermates nor did it affect any C21 steroid.

**Table 1:** Steroid content of testis, prostate, and liver from puberal and postpuberal littermate boars treated with the aromatase inhibitor, letrozole, or with the canola oil vehicle^1^.

Testis^2^						
C18	V-16	L-16	V-20	L-20	V-40	L-40
Estrone	22.07 (9.92)	**1.96** (0.78)	17.03 (8.97)	**1.88** (0.49)	9.96 (2.71)	**4.48** (2.28)
Estrone-3-SO4	76.11 (29.56)	**0.92** (0.45)	50.75 (35.24)	**2.02** (2.44)	49.52 (19.75)	46.42 (35.49)
Estrone-3-GLU	ND	ND	ND	ND	ND	ND
16α-Hydroxyestrone	1.70 (0.68)	**0.04** (0.04)	0.68 (0.08)	**0.13** (0.11)	0.51 (0.31)	0.33 (0.09)
Estradiol	44.45 (29.11)	**7.88** (3.74)	56.26 (27.04)	**7.86** (4.62)	39.59 (4.83)	**19.68** (10.45)
Estradiol-3-SO4	586.5 (246.2)	**2.69** (1.14)	381.6 (271.3)	**8.30** (8.91)	273.9 (70.6)	199.7 (129.9)
Estradiol-3-GLU	0.27 (0.26)	0.12 (0.18)	0.31 (0.62)	0.03 (0.06)	0.02 (0.04)	0.02 (0.03)
Estradiol-17-GLU	3.64 (3.30)	0.66 (0.77)	2.94 (2.18)	0.97 (1.08)	2.13 (2.13)	0.24 (0.38)
Estriol	0.20 (0.29)	trace	0.29 (0.41)	0.01 (0.02)	0.04 (0.08)	0.04 (0.08)
C19	V-16	L-16	V-20	L-20	V-40	L-40
Dehydroepiandrosterone	85.96 (23.65)	132.90 (138.80)	98.91 (105.92)	48.55 (45.99)	52.26 (30.66)	37.13 (32.51)
Dehydroepiandrosterone-SO4	237.8 (43.87)	163.7 (179.6)	193.7 (71.79)	125.9 (70.36)	98.37 (15.06)	102.0 (55.65)
5-Androstenediol	75.40 (35.83)	104.3 (107.7)	86.29 (113.7)	37.4 (30.84)	84.27 (88.43)	28.20 (22.01)
4-Androstenediol	5.58 (4.64)	5.01 (4.81)	5.35 (6.13)	1.89 (1.21)	4.77 (3.41)	2.20 (1.87)
Androstenedione	58.11 (31.33)	36.20 (32.91)	53.49 (32.36)	21.24 (9.45)	18.20 (12.55)	13.08 (4.79)
19-Hydroxyandrostendione	12.80 (14.66)	0.35 (0.34)	10.60 (11.26)	0.93 (0.99)	2.61 (1.42)	3.07 (2.78)
Testosterone	236.2 (130.6)	157.8 (192.4)	166.5 (92.85)	84.42 (59.00)	61.86 (36.59)	36.84 (12.43)
Epitestosterone	4.50 (2.95)	2.42 (3.65)	4.81 (2.94)	2.32 (0.76)	1.01 (0.74)	0.67 (0.49)
Epi/Testosterone-SO4	12.60 (6.59)	11.32 (4.70)	8.44 (6.36)	4.27 (2.42)	4.56 (3.92)	7.53 (6.41)
5α-Dihydrotestosterone	1.87 (1.88)	4.39 (6.76)	2.42 (2.58)	3.12 (3.31)	0.54 (0.63)	1.33 (1.09)
6-Ketotestosterone	59.83 (96.59)	55.22 (75.38)	45.08 (55.70)	33.28 (27.30)	7.44 (11.89)	5.88 (5.80)
6-Dehydrotestosterone	0.86 (0.46)	0.40 (0.25)	0.29 (0.15)	0.30 (0.09)	0.21 (0.12)	0.22 (0.13)
17β-Dihydroepiandrosterone	181.7 (162.6)	174.8 (242.7)	151.0 (170.3)	98.98 (46.79)	130.6 (93.27)	82.13 (51.50)
6α-Hydroxytestosterone	12.16 (8.52)	3.54 (6.53)	9.13 (6.32)	2.02 (3.49)	1.49 (0.90)	0.88 (0.79)
Adrenosterone	2.74 (2.82)	2.37 (2.47)	1.40 (0.89)	1.49 (1.15)	0.39 (0.45)	0.40 (0.29)
Epiandrosterone	12.85 (7.10)	15.66 (26.94)	10.91 (7.72)	10.13 (14.69)	3.81 (4.12)	6.51 (5.76)
C21						
Pregnenolone	835.4 (357.8)	822.5 (462.5)	739.8 (594.0)	806.3 (725.1)	484.4 (107.0)	397.8 (239.8)
Pregnenolone-3-SO4	59.3 (11.5)	130.6 (177.8)	66.7 (52.8)	87.7 (77.3)	39.8 (20.9)	38.5 (33.6)
17-Hydroxypregnenolone	396.0 (265.0)	406.5 (360.5)	421.4 (507.5)	395.9 (536.0)	255.1 (208.4)	214.1 (147.9)
Progesterone	6.92 (2.26)	10.30 (6.50)	9.03 (9.34)	16.86 (23.74)	5.08 (4.11)	4.14 (2.98)
17-Hydroxyprogesterone	2.52 (1.01)	4.04 (2.70)	3.48 (3.44)	4.68 (4.61)	2.89 (2.13)	2.08 (1.08)
11-Deoxycortisol	2.88 (2.15)	1.07 (0.72)	2.25 (1.42)	0.95 (0.71)	0.97 (0.55)	0.49 (0.30)
Cortisol	4.14 (4.13)	0.69 (0.79)	3.27 (3.09)	1.01 (0.99)	2.10 (1.49)	2.09 (1.39)
Deoxycorticosterone	1.59 (1.22)	0.50 (0.43)	1.35 (1.02)	0.44 (0.26)	0.44 (0.35)	0.27 (0.11)
Corticosterone	0.27 (0.52)	0.41 (0.43)	1.02 (1.81)	0.16 (0.12)	0.37 (0.27)	0.42 (0.27)
Epiallopregnanolone	78.13 (62.52)	120.8 (147.3)	81.82 (97.05)	162.6 (179.2)	81.87 (95.14)	47.55 (25.79)
Allo/Pregnanolone-SO4	1.59 (1.36)	3.78 (3.38)	0.93 (0.61)	1.90 (1.84)	0.42 (0.33)	0.23 (0.18)
Epi/Epiallopregnanolone-SO4	2.91 (1.80)	21.39 (35.92)	7.94 (11.73)	14.97 (18.26)	4.37 (5.82)	3.15 (3.72)
Prostate^3^						
C18	V-16	L-16	V-20	L-20	V-40	L-40
Estrone	3.64 (1.86)	1.59 (0.51)	2.14 (0.92)	2.04 (1.18)	2.94 (2.15)	1.65 (0.49)
Estrone-3-SO4	9.37 (7.78)	**0.36** (0.19)	5.27 (2.42)	**0.49** (0.12)	9.32 (8.19)	4.00 (3.01)
Estrone-3-GLU	ND	ND	ND	ND	ND	ND
16α-Hydroxyestrone	ND	ND	ND	ND	ND	ND
Estradiol	18.33 (8.28)	11.80 (5.35)	15.50 (10.56)	13.34 (7.89)	19.43 (14.06)	8.04 (1.98)
Estradiol-3-SO4	7.44 (5.38)	**0.54** (0.42)	4.28 (1.64)	**0.73** (0.99)	10.86 (13.35)	3.25 (1.92)
Estradiol-3-GLU	0.35 (0.57)	0.002 (0.002)	0.05 (0.09)	0.12 (0.17)	1.31 (0.99)	0.02 (0.03)
Estradiol-17-GLU	0.42 (0.72)	0.002 (0.001)	0.20 (0.40)	1.51 (2.00)	1.40 (1.23)	0.94 (0.86)
Estriol	ND	ND	ND	ND	ND	ND
C19						
Dehydroepiandrosterone	ND	ND	ND	ND	ND	ND
Dehydroepiandrosterone-SO4	29.29 (19.57)	9.89 (7.27)	15.36 (5.18)	12.16 (7.53)	31.03 (24.15)	13.15 (10.51)
5-Androstenediol	ND	ND	ND	ND	ND	ND
4-Androstenediol	ND	ND	ND	ND	ND	ND
Androstenedione	0.26 (0.10)	0.24 (0.09)	0.22 (0.10)	0.13 (0.06)	0.38 (0.29)	0.13 (0.01)
19-Hydroxyandrostendione	ND	ND	ND	ND	ND	ND
Testosterone	1.67 (0.32)	1.04 (1.67)	0.36 (0.15)	0.55 (0.29)	1.07 (0.76)	0.33 (0.15)
Epitestosterone	0.01 (0.02)	0.09 (0.07)	0.02 (0.02)	0.09 (0.10)	0.27 (0.53)	0.27 (0.28)
Epi/Testosterone-SO4	ND	ND	ND	ND	ND	ND
5α-Dihydrotestosterone	2.51 (1.10)	1.79 (1.17)	1.29 (0.84)	1.23 (0.39)	0.93 (0.95)	0.99 (0.49)
6-Ketotestosterone	ND	ND	ND	ND	ND	ND
6-Dehydrotestosterone	ND	ND	ND	ND	ND	ND
17β-Dihydroepiandrosterone	trace	trace	trace	trace	trace	trace
6α-Hydroxytestosterone	ND	ND	ND	ND	ND	ND
Adrenosterone	ND	ND	ND	ND	ND	ND
Epiandrosterone	4.76 (2.82)	6.24 (5.94)	6.01 (4.52)	7.01 (4.62)	4.41 (3.25)	3.27 (2.01)
C21						
Pregnenolone	ND	ND	ND	ND	ND	ND
Pregnenolone-3-SO4	0.49 (0.39)	0.36 (0.29)	0.42 (0.19)	1.29 (0.88)	0.98 (0.80)	0.62 (0.55)
17-Hydroxypregnenolone	ND	ND	ND	ND	ND	ND
Progesterone	0.70 (0.34)	0.31 (0.21)	0.73 (0.86)	1.64 (2.36)	0.58 (0.50)	0.25 (0.20)
17-Hydroxyprogesterone	ND	ND	ND	ND	ND	ND
11-Deoxycortisol	ND	ND	ND	ND	ND	ND
Cortisol	2.85 (1.87)	0.86 (0.72)	3.13 (4.05)	3.39 (3.70)	3.38 (3.57)	1.12 (0.69)
Deoxycorticosterone	0.24 (0.36)	0.11 (0.10)	0.24 (0.26)	0.24 (0.25)	0.07 (0.11)	0.08 (0.07)
Corticosterone	1.84 (1.38)	0.36 (0.50)	0.82 (1.15)	2.32 (3.12)	0.26 (0.17)	0.35 (0.28)
Epiallopregnanolone	ND	ND	ND	ND	ND	ND
Allo/Pregnanolone-SO4	ND	ND	ND	ND	ND	ND
Epi/Epiallopregnanolone-SO4	trace	trace	trace	trace	trace	trace
Liver^4^						
C18	V-16	L-16	V-20	L-20	V-40	L-40
Estrone	0.22 (0.03)	0.33 (0.13)	0.53 (0.20)	0.41 (0.05)	0.48 (0.21)	0.29 (0.12)
Estrone-3-SO4	4.84 (3.40)	**0.06** (0.04)	6.02 (1.51)	**0.26** (0.39)	6.85 (4.79)	2.18 (1.30)
Estrone-3-GLU	1.06 (0.36)	0.12 (0.22)	0.14 (0.13)	0.36 (0.44)	1.40 (2.23)	0.90 (1.01)
16α-Hydroxyestrone	0.01 (0.003)	0.03 (0.01)	0.02 (0.01)	0.02 (0.01)	0.02 (0.01)	0.02 (0.004)
Estradiol	1.27 (0.05)	1.68 (0.84)	2.89 (2.07)	1.47 (0.50)	2.40 (0.80)	1.43 (1.06)
Estradiol-3-SO4	14.26 (15.44)	**0.13** (0.08)	34.31 (12.07)	**1.35** (1.70)	67.40 (43.92)	21.61 (14.41)
Estradiol-3-GLU	1.19 (1.33)	**0.004** (0.01)	0.95 (0.88)	0.02 (0.02)	3.05 (3.03)	2.18 (1.72)
Estradiol-17-GLU	1.11 (1.57)	**0.32** (0.64)	0.57 (0.47)	0.13 (0.15)	1.79 (0.29)	0.62 (0.67)
Estriol	0.07 (0.10)	trace	0.16 (0.23)	trace	0.01 (0.02)	0.21 (0.34)
C19	V-16	L-16	V-20	L-20	V-40	L-40
Dehydroepiandrosterone	ND	ND	ND	ND	ND	ND
Dehydroepiandrosterone-SO4	11.14 (4.38)	6.96 (3.49)	16.70 (4.33)	11.66 (10.59)	17.78 (14.61)	9.23 (6.83)
5-Androstenediol	ND	ND	ND	ND	ND	ND
4-Androstenediol	ND	ND	ND	ND	ND	ND
Androstenedione	0.07 (0.01)	0.13 (0.05)	0.34 (0.23)	0.17 (0.08)	0.11 (0.09)	0.07 (0.04)
19-Hydroxyandrostendione	ND	ND	ND	ND	ND	ND
Testosterone	0.19 (0.01)	0.31 (0.27)	0.43 (0.05)	0.65 (0.32)	0.60 (0.49)	0.31 (0.08)
Epitestosterone	0.26 (0.08)	0.18 (0.15)	0.14 (0.11)	0.25 (0.23)	0.20 (0.21)	0.19 (0.19)
Epi/Testosterone-SO4	2.35 (2.00)	2.15 (2.10)	2.87 (0.76)	2.90 (3.31)	4.93 (2.03)	4.79 (4.31)
5α-Dihydrotestosterone	0.04 (0.06)	0.09 (0.10)	0.04 (0.07)	0.01 (0.02)	0.13 (0.15)	0.05 (0.07)
6-Ketotestosterone	ND	ND	ND	ND	ND	ND
6-Dehydrotestosterone	ND	ND	ND	ND	ND	ND
17β-Dihydroepiandrosterone	ND	ND	ND	ND	ND	ND
6α-Hydroxytestosterone	ND	ND	ND	ND	ND	ND
Adrenosterone	ND	ND	ND	ND	ND	ND
Epiandrosterone	ND	ND	ND	ND	ND	ND
C21	V-16	L-16	V-20	L-20	V-40	L-40
Pregnenolone	ND	ND	ND	ND	ND	ND
Pregnenolone-3-SO4	0.60 (0.02)	0.71 (0.86)	0.87 (0.54)	1.07 (0.64)	0.95 (0.39)	0.96 (0.76)
17-Hydroxypregnenolone	ND	ND	ND	ND	ND	ND
Progesterone	0.06 (0.03)	0.17 (0.11)	1.09 (1.16)	1.05 (1.09)	0.30 (0.06)	0.30 (0.14)
17-Hydroxyprogesterone	ND	ND	ND	ND	ND	ND
11-Deoxycortisol	0.14 (0.08)	0.28 (0.25)	2.04 (2.09)	1.87 (1.86)	0.36 (0.27)	0.30 (0.19)
Cortisol	0.47 (0.06)	1.64 (2.25)	13.23 (12.51)	7.73 (8.98)	1.40 (1.14)	1.70 (1.05)
Deoxycorticosterone	0.03 (0.01)	0.04 (0.04)	0.38 (0.33)	0.36 (0.45)	0.11 (0.08)	0.07 (0.05)
Corticosterone	0.20 (0.21)	0.30 (0.26)	0.60 (0.54)	0.86 (0.91)	0.07 (0.06)	0.12 (0.11)
Epiallopregnanolone	ND	ND	ND	ND	ND	ND
Allo/Pregnanolone-SO4	ND	ND	ND	ND	ND	ND
Epi/Epiallopregnanolone-SO4	trace	trace	trace	trace	trace	trace

^1^Values represent means (SD) in ng/g. Values in bold indicate statistically significant differences between letrozole-treated boars and vehicle-treated littermates at the indicated age.

^2^Values for testis tissue at each age are based upon one boar from each of four litters treated with letrozole from 11 to 16 weeks of age and littermates treated with the canola oil vehicle. All boars came from a total of five litters.

^3^Values for prostate tissue at each age are based upon one boar from each of four litters treated with letrozole from 11 to 16 weeks of age (three boars treated with vehicle at 16 weeks of age and three boars treated with letrozole at 20 weeks of age) and littermates treated with the canola oil vehicle. Tissues came from same boars that donated testicular tissue.

^4^Values for liver tissue at each age are based upon one boar from each of four litters treated with letrozole from 11 to 16 weeks of age (three boars treated with vehicle at 16 weeks of age and at 20 weeks of age) and littermates treated with the canola oil vehicle. Tissues came from same boars that donated testicular tissue.

### Testis, prostate, and liver have unique C18, C19, and C21 steroid profiles

**Fig 2:**
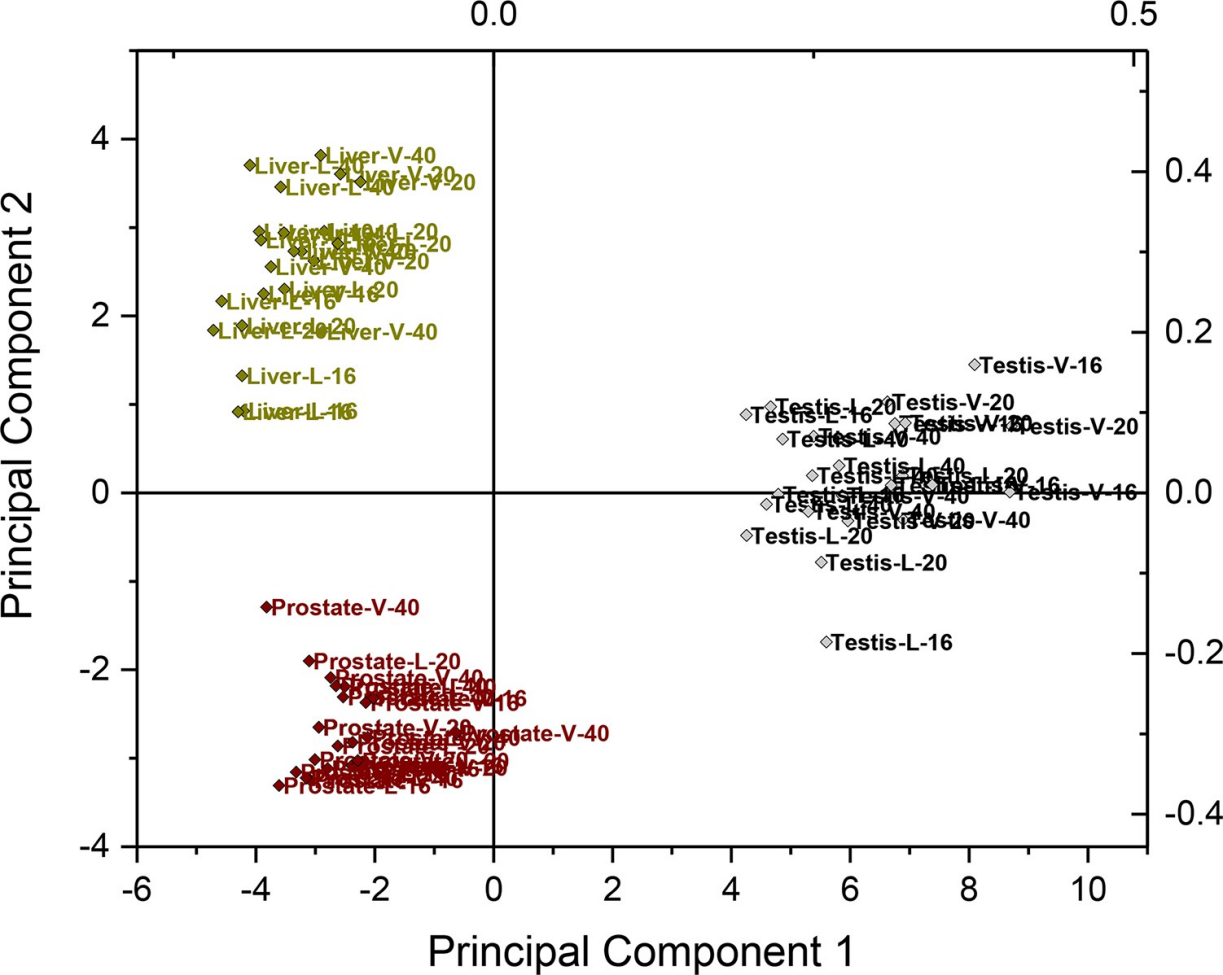
Principal component analysis of steroids in the boar testis, prostate, and liver. The source organ was the primary influence for clustering of steroid profiles rather than age or treatment. The first two of the principal components accounted for 68% of the total variance (54% and 14%) and remaining calculated components each explained 2–7% of the total variance and were not subsequently considered.

**Fig 3:**
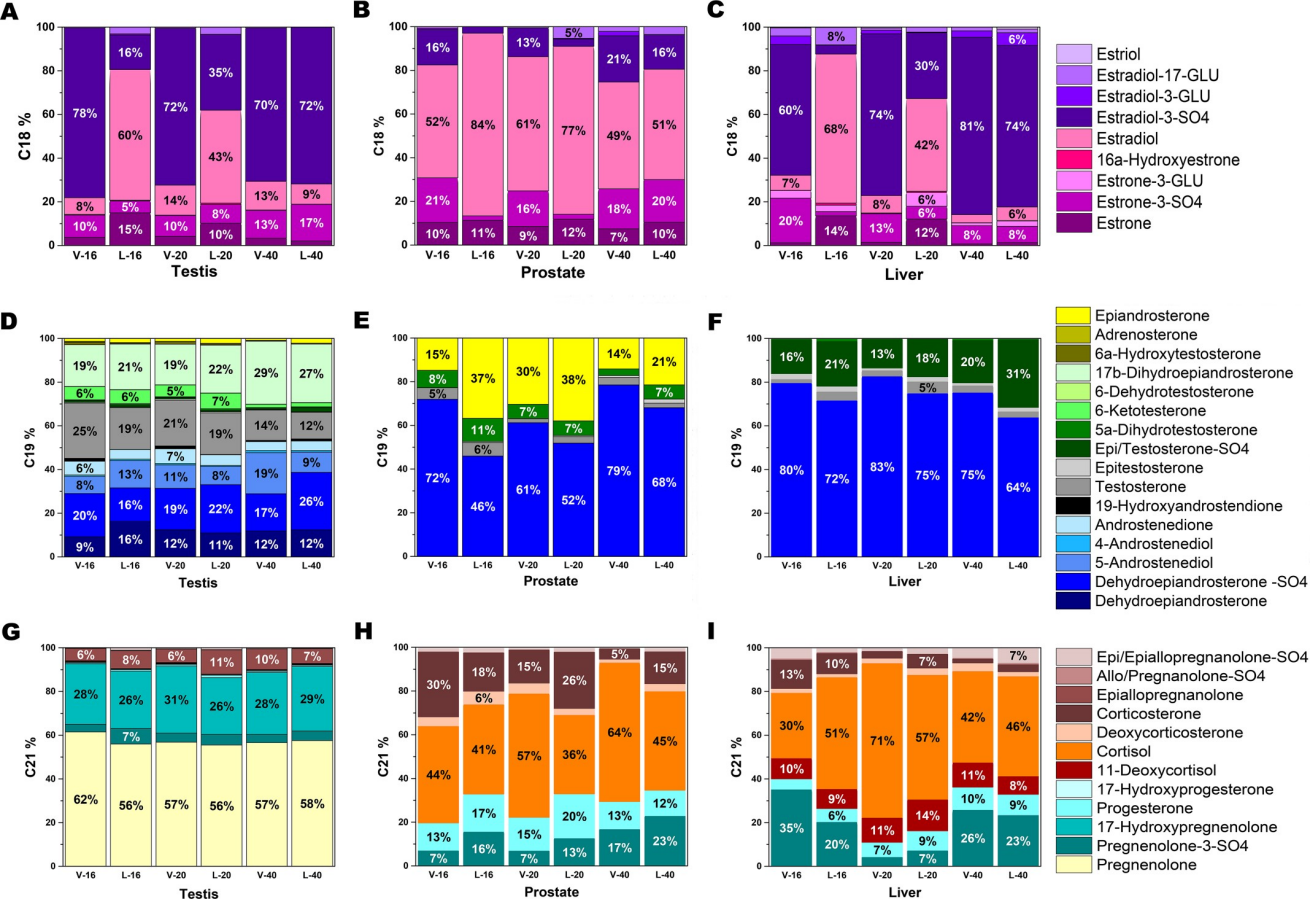
Steroid distributions of C18, C19, and C21 in the testis, prostate, and liver. C18 steroids (**A-C**) consist of estrogen derivatives, C19 steroids (**D-F**) consist of androgen derivatives, and C21 steroids (**G-I**) consist of progestogens, gluco/mineralocorticoids, and derivatives. Each tissue had a distinct profile. C refers to vehicle-treated littermates and L to letrozole-treated littermates. The number following C or L refers to the age of the animal at tissue recovery. Data based on nmol/g tissue.

Each tissue had a unique steroid distribution, which drove the clustering of samples in principal component analysis regardless of treatment group (Figs 2 and 3), although DHEA-sulfate was the predominant C19 steroid hormone in all tissues. The testes had the most diverse steroid profile of the three tissues investigated, containing 40 different identified C18, C19, and C21 steroids (Table 1). This represents a relatively small proportion of the 92 estrogens (C18), 50 androgens (C19), and 60 progestogens and gluco/mineralocorticoids (C21) that might have been detected with this method (Table 2). Quantitatively, the C21 steroids had the largest overall contribution in the testes, followed by C19 and C18 steroids (Table 1). Pregnenolone was the most abundant steroid in boar testis followed by 17-hydroxypregnenolone or estradiol 3-SO4. Pregnenolone levels were twice the concentration of 17-hydroxypregnenolone and five or more fold higher than any other C21 steroid in the testis in all boars. Interestingly, neither pregnenolone nor 17-hydroxypregnenolone was detected in prostate or liver. Pregnenolone-sulfate was detected in all three tissues with concentrations in testis being 20 or more fold higher than concentrations in the prostate and liver.

**Table 2:** List of steroids evaluated in profile.

Steroid	LOD^1^	Steroid	LOD^1^
Pregnenolone	1.58	Estradiol-3-hemisuccinate	0.001
17-Hydroxypregnenolone	1.504	Estrone-3-hemisuccinate	0.007
Dehydroepiandrosterone	0.087	Estradiol-17-hemisuccinate	0.268
5-Androstenediol	0.172	Estradiol-3,17-di-hemisuccinate	0.001
Progesterone	0.003	Estriol-3-hemisuccinate	0.064
17-Hydroxyprogesterone	0.007	Allopregnanolone	*0*.*785*
11-Deoxycortisol	0.007	Alldihydroprogesterone	*1*.*58*
Cortisol	0.068	Allopregnanediol	*0*.*1*
11-Deoxycorticosterone	0.015	11α-Hydroxyprogesterone	*0*.*757*
Corticosterone	0.072	11β-Hydroxyprogesterone	*0*.*076*
Aldosterone	0.693	21-Hydroxypregnanolone	*3*.*008*
Androstenedione	0.009	21-Hydroxypregnenolone	*3*.*008*
Testosterone	0.009	7α-Hydroxypregnenolone	*3*.*008*
5α-Dihydrotestosterone	0.344	2α-Hydroxytestosterone	*0*.*81*
Estrone	0.009	20α-Hydroxy-5a-pregnan-3-one	*0*.*785*
Estradiol	0.009	20α-Hydroxyprogesterone	*0*.*521*
11α-Hydroxyestrone	0.087	17α-20a-Dihydroprogesterone	*0*.*923*
11β-Hydroxyestrone	0.873	Pregnenolone-SO4	0.063
11α-Hydroxyestradiol	0.173	Pregnanolone	*0*.*785*
11β-Hydroxyestradiol	1.734	Pregnanediol	*1*.*56*
9(11)-Dehydroestradiol	0.092	Pregnanedione	*1*.*58*
9(11)-Dehydroestrone	0.093	Epiallopregnanolone	1.36
11-Ketoestrone	0.088	17β-Dihydroandrosterone	*3*.*419*
Estradiol-3-SO4	0.025	17β-Dihydroepiandrosterone	1.71
Estrone-3-SO4	0.076	7α-Hydroxytestosterone	*0*.*821*
Estradiol-17-SO4	*0*.*049*	7α-Hydroxyandrostenedione	*0*.*083*
Estradiol-3,17-SO4	*0*.*216*	6α-Hydroxytestosterone	0.019
3-o-methoxy-estrone	0.009	6β-Hydroxytestosterone	.
Estradiol-3-GLU	1.062	Dehydroepiandrosterone-GLU	*3*.*46*
Estrone-3-GLU	0.073	16α-Hydroxydehydroepiandrosterone	*8*.*55*
Estradiol-17-GLU	1.062	Testosterone-SO4	0.062
Estrone-3,17-GLU	0.327	Desoxytestosterone	*0*.*544*
4-Hydroxyestrone	0.002	Testosterone-GLU	*0*.*166*
4-Hydroxyestradiol	0.347	4-Androsten-3α-ol-17-one	*2*.*19*
4-o-Methoxy-estrone	0.083	2α-Hydroxyandrostenedione	*6*.*22*
4-o-Methoxy-estradiol	3.307	11α-Hydroxyandrostenedione	*1*.*84*
4-o-Methoxy-estriol	15.703	6-Ketotestosterone	0.413
4-Hydroxy-estrone-2-SG	0.042	11-Ketotestosterone	*0*.*928*
4-Hydroxy-estradiol-2-SG	0.169	9-Dehydrotestosterone	.
4-Hydroxy-estrone-2-CYS	0.025	Allotetrahydrocortexone	.
4-Hydroxy-estradiol-2-CYS	0.614	Adrenosterone	0.227
4-Hydroxy-estrone-2-NAcCYS	0.224	Etiocholanolone	.
4-Hydroxy-estradiol-2-NAcCYS	0.223	4-Androstenediol	0.826
4-Hydroxy-estrone-1-N3Ade	0.012	16α-Ketotestosterone	*2*.*21*
4-Hydroxy-estradiol-1- N3Ade	0.024	19-Hydroxyandrostenedione	0.235
4-Hydroxy-estrone-1-N7Gua	0.23	4-Androsten-3,6-17-trione	*2*.*63*
4-Hydroxy-estradiol-1- N7Gua	0.229	7β-Hydroxydehydroepiandrosterone	*6*.*24*
2-Hydroxyestrone	0.349	7α-Hydroxydehydroepiandrosterone	*6*.*22*
2-Hydroxyestradiol	0.347	7-Ketodehydroepiandrosterone	*1*.*12*
2-Hydroxy-estrone-1+4-SG	0.423	9-Dehydroepiandrosterone	.
2-Hydroxy-estradiol-1+4-SG	0.421	Cortisone	.
2-Hydroxy-estrone-1+4-Cys	0.247	9-Dehydroprogesterone	*5*.*29*
2-Hydroxy-estradiol-1+4-Cys	0.614	11-Dehydrotetrahydrocosticosterone	*0*.*814*
2-Hydroxy-estrone-1+4-NAcCys	0.112	Tetrahydrocorticosterone	.
2-Hydroxy-estradiol-1+4-S NAcCys	0.557	6β-Hydroxycorticosterone	*0*.*618*
2-Hydroxyestrone-6-N3Ade	0.119	5α-Dihydrocorticosterone	*1*.*66*
2-Hydroxyestradiol-6-N3Ade	0.119	5α-Dihydropregnanolone	.
2-Hydroxyestriol	0.008	Tetrahydrocortisol	.
3-Methoxy-2-hydroxy-estrone	0.166	Pregnanetriol	*1*.*31*
2-Methoxy-3-hydroxy-estrone	0.008	Allotetrahydrocortisol	.
2-Methoxy-3-hydroxy-estradiol	0.331	16α-Hydroxyprogesterone	*2*.*25*
2,3 Dimethoxy-estrone	0.032	6-Dehydrotestosterone	3.27
2,3 Dimethoxy-estradiol	0.079	9(11)-Didehydroepiandrosterone	*9*.*36*
6α-Hydroxyestradiol	0.173	11β-Hydroxyepiandrosterone	*1*.*28*
6β-Hydroxyestradiol	0.173	16α-Hydroxyepiandrosterone	*8*.*55*
6-Ketoestrone	0.088	Androsterone	.
6-Ketoestradiol	0.087	Epiandrosterone	0.67
6-Dehydroestradiol	0.092	7α-Hydroxyandrostenediol	*1*.*38*
6-Dehydroestrone	0.019	7β-Hydroxyandrostenediol	*1*.*29*
16α-Hydroxyestrone	0.175	5β-Dihydrocorticosterone	.
17-Epiestriol	0.173	4-Pregnen-3b-ol-20-one	*8*.*06*
Estriol	0.347	Urocortisone	*0*.*067*
16,17-Epiestriol	3.468	5-Androsten-3b,17-diol-16-one	*3*.*94*
16-Epiestriol	3.468	19-Hydroxydehydroepiandrosterone	*2*.*8*
Estriol-3-SO4	0.271	16α-Hydroxypregnenolone	*4*.*401*
Estriol-3-GLU	2.153	5α-Dihydrocortexone	*1*.*62*
3-Methoxy-estriol	0.002	Epitestosterone	0.174
16-Keto-17β-estradiol	3.492	5β-Androstanedione	.
16-Ketoestriol	0.331	11β-Hydroxyandrostenedione	.
7-Dehydro-17β-estradiol	3.699	11-Ketoetiocholanolone	.
Equilin	0.019	5α-Pregnan-11a-ol-3,20-dione	*2*.*51*
Dihydroequilin-3-SO4	1.342	5α-Epoxypregnenolone	.
Equilin-3-SO4	6.749	Allopregnanetrione	.
Estrone-9-N3-Ade	0.124	11-Ketoprogesterone	*3*.*12*
Estradiol-9-N3-Ade	0.247	11-Dehydrocorticosterone	.
Estradiol-3-acetate	0.08	6β-Hydroxycortisol	.
Estrone-3-acetate	0.16	20β-Dihydrocortisone	.
Estradiol-3,17α-di-acetate	0.07	6β-Hydroxycortisone	.
Estradiol-3,17β-di-acetate	1.402	Etiocholanolone-GLU	*0*.*398*
2-Hydroxy-estradiol-17-acetate	0.003	Dehydroepiandrosterone-SO4	0.198
6-Ketoestradiol-3,17-di-acetate	0.006	Epitestosterone-SO4	0.181
6-Ketoestradiol-tri-acetate	0.023	Epitestosterone-GLU	*0*.*126*
6-Dehydroestradiol-di-acetate	0.141	Pregnanediol-GLU	*0*.*0183*
Estriol-3-acetate	0.303	17α-Hydroxypregnanolone-GLU	*1*.*76*
Estriol-16-acetate	0.303	Pregnanolone-SO4	0.133
Estriol-16,17-diacetate	0.268	17α,20β-Dihydroxyprogesterone-GLU	*0*.*102*
17-Epiestriol triacetate	0.241	Hydrocortisone-21-SO4	.
Estriol-triacetate	0.006	Pregnenolone-GLU	.
16,17-Epiestriol triacetate	0.06	Allopregnanolone-SO4	0.014
16-Epiestriol triacetate	0.121	Epiallopregnanolone-SO4	0.012
Equilin acetate	0.081	Corticosterone-21-SO4	.

^1^LOD values are estimates of the limit of detection. Values in italics are based on a limited assessment and no estimates are available for compounds with a period.

**Fig 4:**
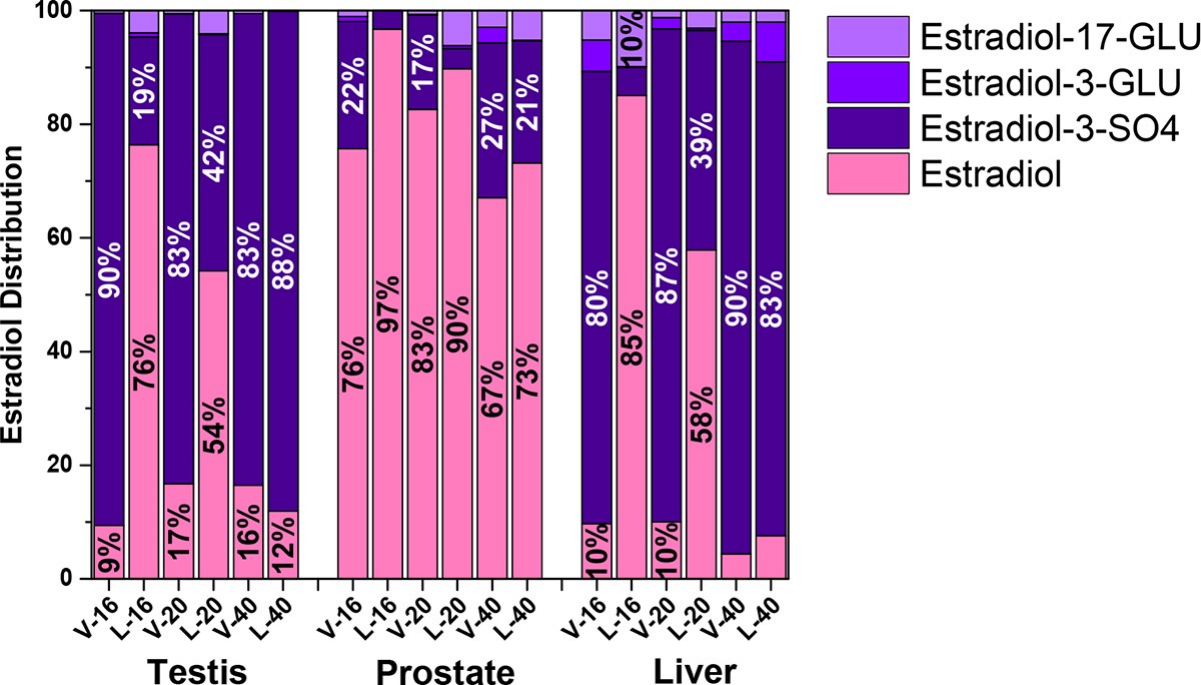
Distribution of estradiol and estradiol conjugates in testis, prostate, and liver. Estradiol was the predominant C18 steroid in each of these tissues. The prominence of estradiol sulfate in testis and liver from vehicle-treated boars contrasts with the limited concentration of the sulfo-conjugate in the prostate, even in vehicle-treated boars. A shift in distribution between free estradiol and estradiol sulfate is apparent in the 16 and 20 week letrozole-treated littermates. C refers to vehicle-treated littermates and L to letrozole-treated littermates. The number following C or L refers to the age of the animal at tissue recovery.

The prostate contained 20 different identified C18, C19, and C21 steroids (Table 1). Estradiol or DHEA-sulfate was the most abundant steroid in the prostate. Regardless of treatment, total C21,C19,C18 steroid concentrations in the prostate were ~50% C18, ~50% C19, and less than 10% C21 (Table 1). Total C18 steroids in letrozole-treated animals were less than half the concentration detected in the testis in vehicle-treated littermates. Most of the C18 steroids were present in an unconjugated form in the prostate in contrast to the conjugated form in the testis (Fig 4). Only six different C19 steroids were detected in the prostate compared with 17 detected in the testis. Although most C19 steroids were markedly lower in the prostate than the testis, 5α-dihydrotestosterone had a similar concentration in both tissues.

The liver contained 23 different identified C18, C19, and C21 steroids. The C18 steroids quantitatively accounted for over 50% of total steroids in vehicle-treated boars and less than 20% of the total steroids in the liver from the 16 and 20 week letrozole-treated boars consistent with reduced production of C18 steroids in these boars (Table 1). Regardless of treatment, steroid levels in liver tissue were much, much lower than in the testis and total free steroid concentration was much lower than in the prostate.

### Prostate enzymes

Aromatase activity in the prostate was detectable but almost 1000 fold lower than that in the testes of the same animals (0.08 vs 65.20 pmoles/mg protein/h, standard error = 0.27, P < 0.001). Concentrations of the aromatase precursors, testosterone and androstenedione were quite low in the prostate (less than 2% of testicular levels) and not affected by letrozole treatment. Measurable aromatase activity was not detectable in liver tissues from 16 week boars. In contrast, gene expression for steroid sulfatase (*STS*), the enzyme involved in removing sulfate, was detectable in prostate and testis and liver tissues with similar relative gene expression (ΔCt of 2.88, 1.86, and 2.0 respectively, P > 0.25, standard error = 0.92).

## Discussion

To our knowledge, this study provides the most comprehensive assessment of steroid tissue concentrations in the boar to date. Although the steroids evaluated were extensive, the pheromone androstenone was not included in the panel. Testicular concentrations detected in this study were consistent with the testicular origin indicated for previously described steroid sulfates [41]. In contrast to a previous suggestion that dihydrotestosterone increased from puberty to maturity [42], our data do not suggest an increase in dihydrotestosterone in either the testis or the prostate although our data did not include comparisons with prepuberal timepoints. Apparent age-related decreases in testicular steroids among vehicle-treated boars may reflect increased testicular mass, due at least in part to increased spermatogenesis. Our multi-tissue approach in combination with a broad steroid profile provide critical information on the role of each of these tissues in steroidogenesis, steroid intracrinology, and steroid sulfate metabolism.

### Endogenous estrogen synthesis and metabolism

Letrozole administration uniquely affected endogenous C18 steroids in the testis, prostate, and liver, revealing tissue-specific regulation of estrogen synthesis and synthetic pathways. Testicular estrogens in the boar are primarily synthesized locally via aromatization of androgen precursors [27, 41, 43]; testicular estrogen sulfates result from subsequent local conjugation by sulfotransferases [27, 29, 30]. Letrozole inhibited testicular aromatase, resulting in the direct decrease in estrone and estradiol concentrations and an indirect decrease in the corresponding testicular sulfo-conjugate concentrations. A decrease in 19-hydroxyandrostenedione, an intermediate of aromatase action on androstenedione, might be expected. Although values for 19-hydroxyandrostenedione were numerically much lower in letrozole-treated boars than in vehicle-treated littermates, values were not significantly different, perhaps due to high variability among boars. Letrozole administration during the prepuberal interval had a prolonged effect on estrogen production in this study similar to the reprogramming of aromatase including reduced protein and enzymatic activity observed long after letrozole administration ceased during the neonatal and juvenile intervals [44].

Estrogen sulfate and free estrogen concentrations were affected differently by letrozole administration in the prostate and liver; concentrations of free estrogens were unaffected by letrozole treatment while estrone-sulfate and estradiol-sulfate concentrations were significantly decreased in tissue from letrozole-treated boars. Consistent with the high levels of sulfatase present in the prostate [7, 45], *in vitro* evidence suggests estrone and estradiol are produced in greater quantities in the human prostate by sulfatase than by aromatase [46]. Similarly, the very, very low aromatase activity we detected in the prostate in conjunction with gene expression for steroid sulfatase, the enzyme that removes sulfate from sulfated steroids, suggests local transformation from estrogen sulfates may maintain free estrogen levels in the boar prostate as well. Higher estradiol concentrations in the prostates from 16 and 20 week letrozole-treated boars than concentrations in the testes from these same boars are consistent with this suggestion of local transformation in the prostate distinct from testicular regulation. The significant decrease in estrogen sulfates in the prostate may reflect desulfation of testicular-derived estrogen sulfates in order to maintain free estrogen concentrations in the prostate. Furthermore, prostate intracrine metabolism may enable independent regulation/synthesis of estrogens and androgens via separate testicular precursors, estrogen sulfate and DHEA-sulfate, respectively.

Dependence of boar prostate size on estrogens and androgens was previously addressed by Booth et al. [47–49]. Castrated boars were treated with dihydrotestosterone, DHEA, estrone, or estradiol and prostate size along with other secondary sex glands were monitored. Only males administered estrone or estradiol showed significantly increased mass of prostate, seminal vesicles, and bulbourethral glands [47, 48], confirming that these accessory sex glands are dependent on estrogens. Interestingly, castrated boars administered DHEA, did not have detectable levels of free estrogens and did not have an increased prostate mass [47]. This finding suggests that DHEA is unlikely to be a major estrogen precursor in the prostate consistent with primarily aromatase-independent synthesis of estrogens in the prostate. Our data extend these previous findings suggesting that the porcine prostate primarily synthesizes free estrogens from testicular estrogen sulfates, rather than from DHEA similar to the role of sulfatase proposed in the human prostate [9]. These results are also consistent with use of sulfatase inhibitors to inhibit local synthesis of active estrogens in prostate cancer therapy [46, 50, 51]. Hence, although free estrogen concentrations are significantly decreased in the primary steroidogenic tissue, testes, and systemic circulation [32], free estrogens in a peripheral tissue, the prostate, were not affected. The ability of the prostate to function independently of testicular and systemic influences strongly suggests that future steroid-hormone therapeutic approaches should account for the contribution of steroid sulfates and sulfatase in steroid hormone production in a tissue.

Historically, sulfated steroid hormones in the systemic system were considered metabolic end products, primarily synthesized in the liver, that aid in excretion [52]; although research has challenged and abandoned conceptions of this simple biological role and exclusive origin of steroid sulfates [3, 7, 53]. Based on the current study, the boar liver likely does not drive or significantly impact systemic estrogen sulfate concentrations but likely functions to aid in excretion of circulating estrogens. The expression of estrogen receptors [54] and estrogen-induced hepatocyte regeneration [55] suggest that liver can be a target for estrogens. Furthermore, the liver possesses metabolic machinery to both conjugate and de-conjugate estrogens [7, 27, 56], providing the ability to regulate estrogen concentrations and distribution of free estrogens independent of circulating concentrations. However, free estrogen levels were very low in the liver, suggesting minimal signaling.

Sulfated steroids in tissues can be altered after tissue collection by sulfatase enzymatic activity [30]. However, homogenization of the frozen tissue in ice-cold methanol in the present study would be expected to denature these enzymes prior to significant conversion. The ratios of sulfated to free steroids in the present study are consistent with postharvest inhibition of sulfatase activity in the testis [30].

### Progestogen and gluco/mineralocorticoid synthesis and metabolism

The predominance of C21 steroids detected in the testis is consistent with the testis as a primary endocrine gland in the boar. Pregnenolone and 17-hydroxypregnenolone were prominent in the testis and most likely function as intermediary compounds in testicular steroidogenesis [57]; neither steroid is secreted in quantitatively significant amounts [26]. Endogenous estrogens and androgens can be synthesized metabolically from pregnenolone via either the Δ^5^- or Δ^4^- pathway in the boar [28, 58]. Metabolites in testis extract are consistent with predominance of the Δ^5^- pathway in the testis and support presence of both pathways. These results are consistent with the testis being the primary site for synthesis of systemic DHEA-sulfate [59, 60] as it is in most mammals other than primates [61].

Production of steroid hormones in the prostate likely result from precursor steroids provided systemically. The C21 steroid precursors, pregnenolone, 17-hydroxypregenenolone, and 17-hydroxyprogesterone, were not detectable suggesting they are not components of steroid synthesis within the prostate. Other steroid components of the Δ^5^- pathway were similarly not detectable in the prostate or liver and other components of the Δ^4^- pathway were not detectable or present in only minimal quantities. The lack of substantial free C21 steroids and paucity of the Δ^5^- or Δ^4^- pathway metabolites, support the hypothesis that the boar prostate and liver utilize testis and/or adrenal derived steroids for local steroid hormone synthesis [7, 27] and contrasts with the abundance of these C21 steroid precursors in primary endocrine glands like the testis.

## Conclusion

A multi-tissue approach in combination with aromatase inhibition and investigation of a broad array of steroids, revealed complexity and unique roles for individual organs in steroid hormone synthesis. Results from animals treated with letrozole displayed the primary role of the testis in synthesis of estrogens and estrogen sulfates, and suggested the dependence of the prostate on sulfated testis-derived steroid hormone precursors. Furthermore, individual organs may maintain tissue-specific profiles of active steroid hormones independent of systemic influences. Our research sheds light on the critical regulatory role of steroid sulfates in a tissue and suggests future research on the interplay of free and sulfo-conjugated steroid hormones within a tissue.
